# Description and ecology of two new species of *Gyronotus* van Lansberge, 1874 (Coleoptera, Scarabaeidae) from southern Africa

**DOI:** 10.3897/zookeys.344.6101

**Published:** 2013-10-22

**Authors:** Philippe Moretto, Renzo Perissinotto

**Affiliations:** 1 936 Avenue des Fils Marescot, 83200 Toulon, France; 2 Zoology Department, Nelson Mandela Metropolitan University, P.O. Box 77000, Port Elizabeth 6031, South Africa

**Keywords:** Scarabaeinae, new species, threatened genus, South Africa, Swaziland, montane grassland, sour bushveld, coastal sourveld

## Abstract

Recent collections in KwaZulu-Natal and Swaziland have led to the discovery of two new species of the flightless and highly threatened Scarabaeinae genus *Gyronotus* van Lansberge, 1874. A description of *G. perissinottoi* **sp. n.** and *G. schuelei* **sp. n.** is provided here, along with notes on their habitat and ecology. Unlike the vast majority of the species previously known in the genus, which have been reported as forest dwellers, the two new species are found during daytime in grassland/savanna vegetation, at the margin of forest patches.

## Introduction

The genus *Gyronotus* currently includes six species, subdivided into three species groups on the basis of their biogeographic distribution, body shape and structure of male genitalia (Davis et al. 2008). They are all wingless, with fused elytra, and are regarded among the most endangered of the African Scarabaeinae because of their sensitivity to disturbance. All species described so far, apart from *Gyronotus glabrosus* which is an upland grassland dweller (A. Davis pers. comm.), are linked to coastal and low-lying forest habitats, which have undergone massive transformation during the past 50–100 years, through clearance, degradation and fragmentation (Davis et al. 1999, Mucina and Rutherford 2006). *Gyronotus fimetarius* Kolbe, 1894, and *Gyronotus carinatus* Felsche, 1911, have disappeared from large parts of their original distribution range, namely the East Usambara Mountains of Tanzania and south of Maphelane in KwaZulu-Natal, respectively (Davis et al. 2008).

On the basis of the wide gaps existing between the distribution ranges of the species currently described, Davis et al. (2008) correctly predicted that new records and species would be discovered in the future. Here, we report on two new species that have only recently being recognised and collected in sufficient number to warrant their description. In the case of *Gyronotus perissinottoi* sp. n., ecological observations have also been possible, through repeated survey exercises. This species occurs in a small but biodiversity unique area in southern KwaZulu-Natal. The first female specimen was collected already in 2004 and a second one in 2007, but it was only in January 2013 that a full series including males was finally secured, through intense search and trapping efforts. The second species, *Gyronotus schuelei* sp. n., originates from western Swaziland and is currently known only from two specimens, one male and one female, collected in different places and on separate occasions.

## Methods

As part of the collecting efforts, ground traps baited with fresh baboon dung were deployed in the Umthamvuna Reserve during December 2012 and January 2013. Traps consisted of 600 ml plastic jars of 80 mm mouth diameter and 120 mm height buried in the soil with the top levelled with the ground. They were checked at regular intervals of 2–5 days and after each visit traps were first emptied and then re-supplied with fresh dung.

The description of morphological characters follows the terminology used by Davis et al. (1999). Specimen length was measured from the anterior margin of the clypeus to the apex of the pygidium. Specimen width represents the maximum width of the elytra. Photos of the holotypes and allotypes were taken using a Canon EOS 400D camera fitted with a Canon MP-E 65 mm objective. Photos were processed with photo stacking technique, using Combine ZP (freeware free software by Alan Hadley, http://www.hadleyweb.pwp.blueyonder.co.uk). The first author compiled the taxonomic part of this study, including the descriptions of the new species. The second author collected all the specimens, with one exception, and contributed all habitat description and ecological observations.

Collections are abbreviated as follows: TMSA, Ditsong National Museum of Natural History (formerly Transvaal Museum), Pretoria, South Africa; ISAM, Iziko South African Museum, Cape Town, South Africa; PMOC, Collection Philippe Moretto, Toulon, France; PCRP, Private Collection R. Perissinotto & L. Clennell, Port Elizabeth, South Africa. Geographical abbreviations are as follows: KZN, KwaZulu-Natal Province, South Africa; SWA, Swaziland.

## Taxonomic account

### Gyronotus perissinottoi

Moretto sp. n.

http://zoobank.org/652366C0-B9E4-4526-B217-BB757A0EB0CF

http://species-id.net/wiki/Gyronotus_perissinottoi

Figures 1 , 3

#### Type locality.

South Africa, KwaZulu-Natal, Umthamvuna Nature Reserve, Beacon Hill Section (31°00'47"S, 30°10'23"E); on escarpment at the edge of riverine forest, in grassland interspersed with rocky outcrops and boulders.

#### Type specimens.

Holotype ♂: South Africa, KZN, Umthamvuna Reserve, 13.I.2013, ground trap baited with baboon dung, R Perissinotto & L Clennell legit (TMSA). Allotype ♀: same data as above (TMSA). Paratypes, 3♂ 4♀: same data as above (ISAM, PCPR, PMOC). 1 ♀, same locality and collectors, but 24.I.2004 (PMOC); 1 ♀, same locality and collectors, but 28.I.2007 (PMOC).

#### Diagnosis.

*Gyronotus perissinottoi* sp. n. is, with *Gyronotus dispar* Felsche, 1911, one of the largest *Gyronotus* species currently known. In contrast to all the other species, including *Gyronotus marginatus* Péringuey, 1888, currently synonymized with *Gyronotus pumilus* (Boheman, 1857), the elytra are punctate and the interstriae very marked. Another typical character is the brush of long, flat-laying, yellow setae that cover a narrow area along the middle of the posterior side of the mesofemora in the male. The shape of the parameres is also unique, and closest to that of *Gyronotus pumilus* (*sensu* Scholtz and Howden 1987), with parameres flattened laterally and crossing each other.

#### Etymology.

This species is named after Renzo Perissinotto, who collected the entire type series and compiled the ecological notes reported in this work.

#### Description.

Length 16–18 mm, width 9–10.5 mm; body convex, rounded, shining bronze with metallic reflections, glabrous on the upper side; with few inconspicuous setae on the posterior quarter of elytra in very fresh specimens.

*Head*. Entirely, densely and finely punctate, except the anterior part of the clypeus which is sparsely and very finely dotted; clypeo-genal suture faint; only short segment of occipital suture present on each side of the head, reaching the extremity of the clypeo-genal suture.

**Figure 1.**
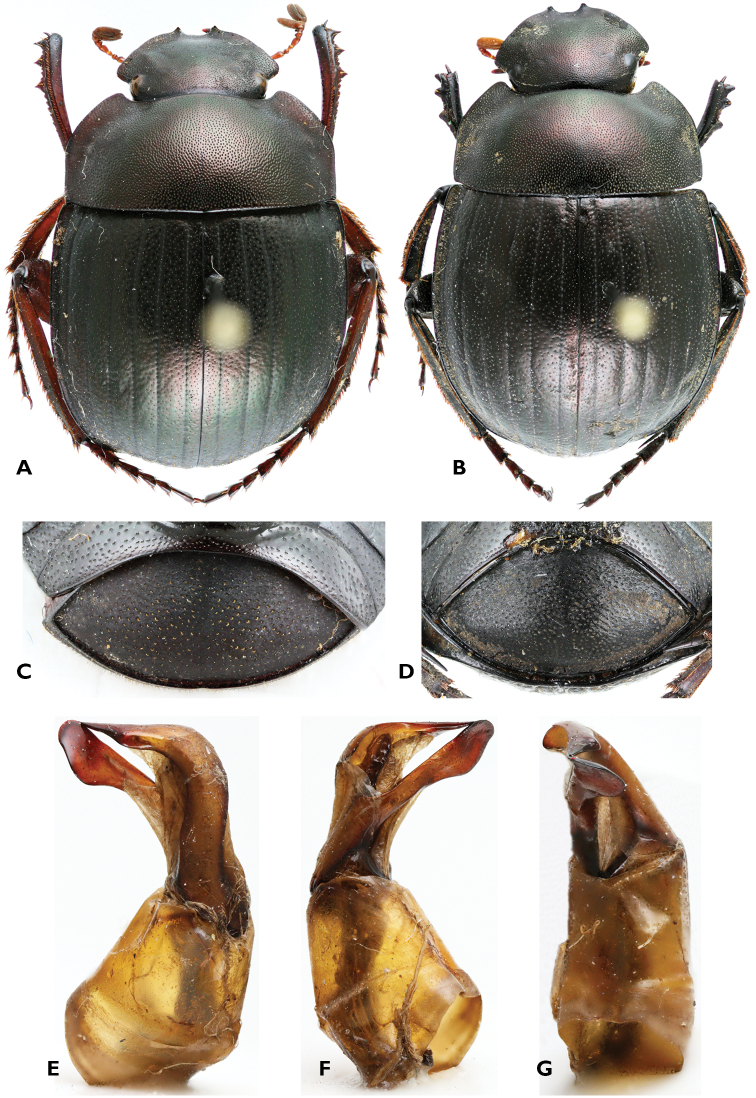
*Gyronotus perissinottoi* sp. n. **A** Holotype male, dorsal habitus **B** Allotype female, dorsal habitus **C** Male pygidium, ventral side **D** Female pygidium, ventral side **E** left **F** right and **G** ventral sides of aedeagus. Photo: Mickaël François.

*Thorax*. Entirely, densely and finely punctate, with punctures finer anteriorly and larger laterally. Lateral angle at middle of sides widely rounded; mesosternum strongly punctate near the suture and in the middle, but finely punctate laterally; metasternum shiny and very finely punctate in the middle part, shagreened and more strongly punctate laterally, with punctures elongated; internal end of male protibia broadened, densely fringed at apex with very short setae, mobile spur short and directed obliquely outwards; female protibia with sharp tooth at the apex of the inner border, mobile spur about twice as long as in the male and directed forward; femora densely punctate; brush of long, flat-laying, yellow setae covering a narrow area along the middle of the posterior side of the mesofemora in male (Figure 1A); brush of setae absent in female (Figure 1B).

*Elytra*. Regularly and strongly convex dorsally, slightly convex laterally; striae obsolete, but densely and strongly punctate, visible with naked eye; interstriae slightly convex, sparsely and finely punctate, with punctures of different size on a very finely shagreened tegument, interspersed with very small, flat and shiny granules.

*Abdomen*. Sternites smooth, finely punctate medially, stronger laterally; pygidium convex, strongly shagreened, strongly and very densely punctate in females, but sparsely and finely with punctures elongated in males; parameres of aedeagus asymmetrical (Figures 1E–G).

#### Remarks.

It was previously known that *Gyronotus pumilus* also occurs in the Umthamvuna Nature Reserve (Davis et al. 2008; pers. observ.). However, the two species occupy different habitats, with *Gyronotus pumilus* restricted to the riverine forest and *Gyronotus perissinottoi* to the grassland plateau, outside the forest.

### Gyronotus schuelei

Moretto sp. n.

http://zoobank.org/8C6B0DE7-D83F-42A3-A35F-FD5EB536A18C

http://species-id.net/wiki/Gyronotus_schuelei

Figures 2

#### Type locality.

Western Swaziland (26°08'18"–26°29'33"S; 31°08'13"–31°11'02"E); in mountain grassland with pockets of sour bushveld.

#### Type specimens.

Holotype ♂: Swaziland, Mlilwane, 28.III.1997, R. Perissinotto & L. Clennell legit (PMOC). Allotype ♀: 19/20.XI.2001, Swaziland, Malolotja Nature Reserve, P. Schüle legit (PMOC).

#### Diagnosis.

This species shows its closest affinity to *Gyronotus glabrosus* Scholtz & Howden, 1987 (p. 84, fig. 3), with which it shares the same structure of the parameres, although their shape is clearly different. In particular, the right paramere is much slender and more regularly curved than its left counterpart, while the apical plate of the left paramere is larger than that on the right.

#### Etymology.

This species is dedicated to Peter Schüle, German specialist of Cicindelidae, who collected the allotype specimen.

#### Description.

Length 14 mm, width 8.5 mm; body moderately convex, very dark brown, with few short, erected, white setae on the posterior half of the elytra.

*Head*. Entirely, densely and finely punctate except for a small space between the clypeal teeth, which is finely dotted.

*Thorax*. Entirely, densely and very finely punctate; exhibiting lateral angle before the middle of the sides; mesosternum strongly but sparsely punctate; metasternum shiny, very finely and sparsely punctate; profemur densely punctuate; mesofemur punctate in the middle, sparsely dotted distally and apically; metafemur sparsely dotted distally, densely punctuate apically.

*Elytra*. Striae obsolete on the disc, more distinct on the sides, finely punctate; interstriae flat on the disc, more convex apically and laterally, finely punctate.

**Figure 2.**
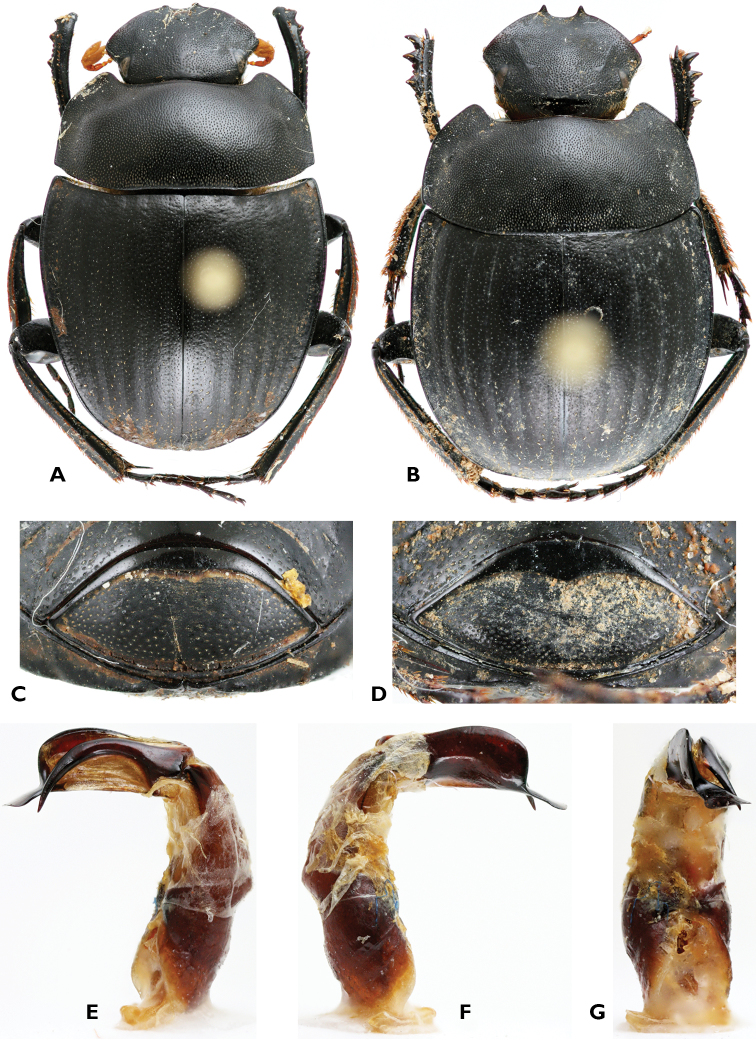
*Gyronotus schuelei* sp. n. **A** Holotype male, dorsal habitus **B** Allotype female, dorsal habitus **C** Male pygidium, ventral side **D** Female pygidium, ventral side **E** left **F** right and **G** ventral sides of aedeagus. Photo: Mickaël François.

*Abdomen*. Pygidium convex in male, totally inflexed in ventral position in female; with raising contour at margin, particularly enlarged at apex in male (Figure 2C), very enlarged in female (Figure 2D); parameres of aedeagus asymmetrical (Figures 2E–G).

**Figure 3.**
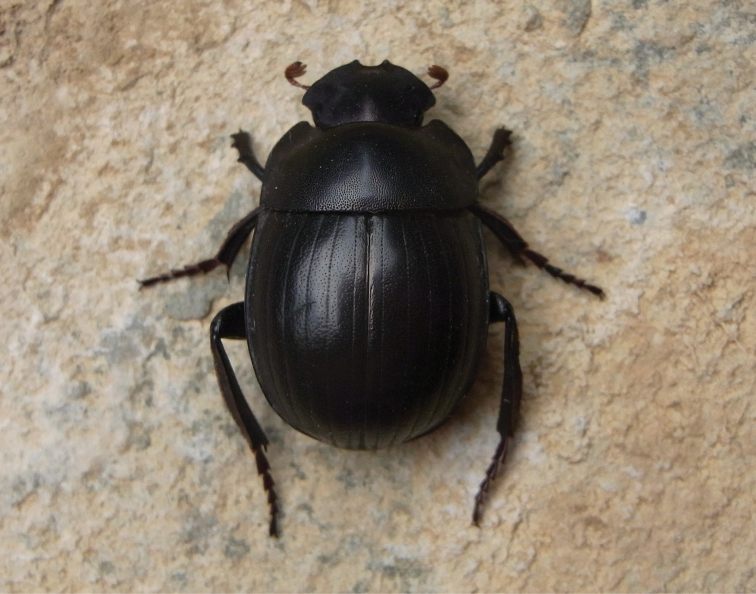
*Gyronotus perissinottoi* sp. n. in its natural habitat at the Umthamvuna Nature Reserve. Photo: Lynette Clennell.

#### Remarks.

This appears to be the first record of the presence of *Gyronotus* in Swaziland. Its distribution range needs to be investigated further, as it may include adjacent mountainous areas in the Mpumalanga Province of South Africa.

## Discussion

The genus *Gyronotus* is part of the tribe Canthonini, which has long been recognised as a Gondwanaland relict (Halffter 1974; Davis et al. 1999). Members of the genus are also wingless and particularly vulnerable to environmental disturbance (Davis et al. 2008). Thus, they are undoubtedly of substantial biodiversity and conservation value, with status ranging from vulnerable to critically endangered (Davis et al. 1999). Much of their recent demise has been attributed to the rapid disappearance of their predominantly forest habitats (Low and Rebelo 1996; Mucina and Rutherford 2006). Indeed, five of the six species described prior to this study are regarded as strictly forest specialists, occurring in coastal and low-lying montane forests on the seaboard of southern and east Africa (Davis et al. 2008).

**Figure 4.**
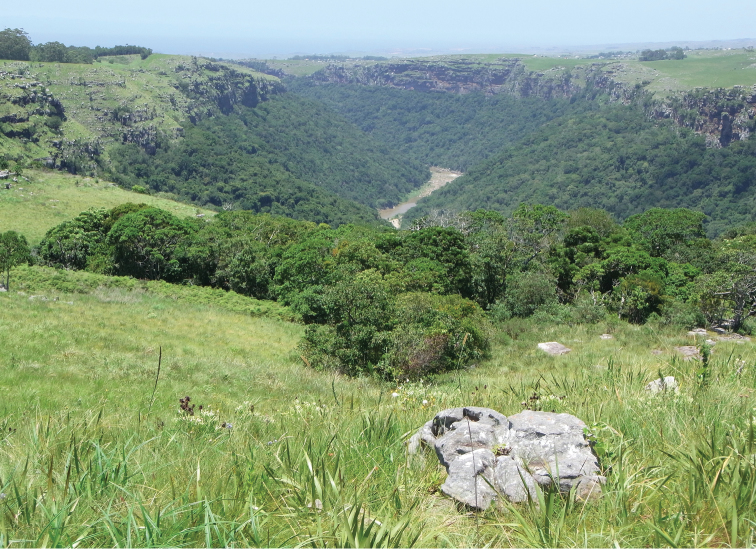
*Gyronotus perissinottoi* sp. n. Typical habitat in the escarpment grassland at the margin of the riverine forest (Umthamvuna Nature Reserve, South Africa). Photo: Lynette Clennell.

The two new species described here, *Gyronotus perissinottoi* sp. n. and *Gyronotus schuelei* sp. n., together with *Gyronotus glabrosus*, are actually grassland or savanna inhabitants. The first is found exclusively in open coastal sourveld with scattered shrubs and small trees, on the plateau and escarpment just above the scarp forest that characterise the Umthamvuna River Gorge, at altitudes of 300–400 m (Figures 3–4). According to the classification scheme of Mucina and Rutherford (2006), this is part of vegetation unit CB4 known as Pondoland-Ugu sandstone coastal sourveld. This is a grassland type that, while exhibiting a large plant diversity and several local endemics, is also among the six most vulnerable vegetation units in South Africa (Mucina and Rutherford 2006). The scarp forest just below, within the gorge, constitutes the typical habitat of *Gyronotus pumilus*.

**Figure 5.**
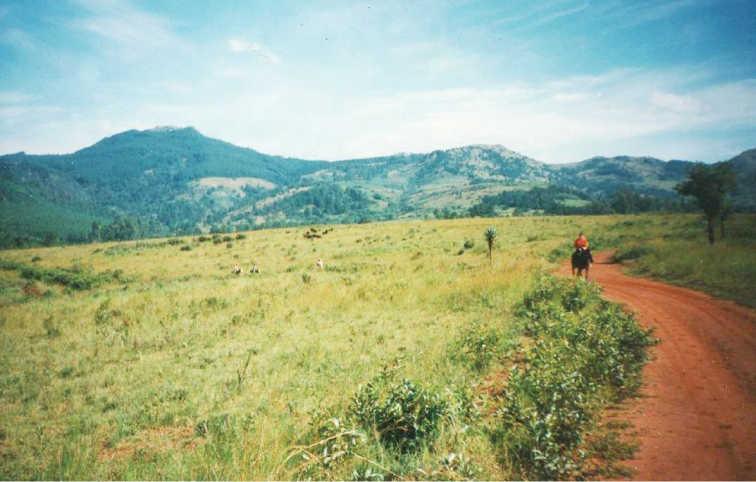
*Gyronotus schuelei* sp. n. Typical habitat in montane grassland interspersed with bushveld pockets (Mlilwane Nature Reserve, Swaziland). Photo: Lynette Clennell.

The Mlilwane and Malolotja reserves of western Swaziland, where *Gyronotus shuelei* sp. n. has been recorded so far, are higher altitude (700–1500 m) hilly areas (Figure 5) that exhibit vegetation units of the KaNgwane montane grassland and Swaziland sour bushveld types, coded respectively Gm 16 and SVI 14 in Mucina and Rutherford (2006). Here, the short and closed grassland layer includes many forbs and scattered shrubs on and around the rocky outcrops. The savanna component consists of a generally open, medium-tall tree layer over a closed, well-developed grass layer. These vegetation units are only regarded as “vulnerable” at present, as a reasonable proportion of the total area is under statutory conservation but much of the remaining part has already been converted to plantations of alien trees or cultivation (Mucina and Rutherford 2006).

Concerning food exploitation, at Umthamvuna *Gyronotus perissinottoi* sp. n. appears to rely mainly on Chacma baboon (*Papio hamadryas*) dung, while *Gyronotus pumilus* inside the forest is regularly observed in or on bushpig (*Potamochoerus larvatus*) dung (RP pers. observ.). The vast majority of *Gyronotus perissinottoi* sp. n. specimens were trapped among rock boulders and outcrops scattered within the grassland, suggesting the possibility that adults may actually prefer to hide dung pellets with eggs under rocks or in crevices, rather than bury them deep into the soil.

No information was unfortunately collected regarding food preferences in *Gyronotus schuelei* sp. n., but the specimen collected at Mlilwane was found during daytime on unidentified herbivore dung. Davis et al. (2008) suggested that species of the genus *Gyronotus* may be day-active. This is confirmed by observations made during the present study, as both species described here as well as *Gyronotus pumilus* were collected while active on the ground during daytime.

## Supplementary Material

XML Treatment for Gyronotus perissinottoi

XML Treatment for Gyronotus schuelei
